# Sport and longevity: an observational study of international athletes

**DOI:** 10.1007/s11357-024-01307-9

**Published:** 2024-08-12

**Authors:** Abdullah Altulea, Martijn G. S. Rutten, Lex B. Verdijk, Marco Demaria

**Affiliations:** 1 European Research Institute for the Biology of Ageing (ERIBA), University Medical Center Groningen (UMCG), University of Groningen (RUG), Groningen, The Netherlands; 2 Department of Human Biology, NUTRIM Institute for Nutrition and Translational Research in Metabolism, Maastricht University, Maastricht, The Netherlands

**Keywords:** Sport, Longevity, Lifestyle

## Abstract

The human lifespan is influenced by various factors, with physical activity being a significant contributor. Despite the clear benefit of exercise on health and longevity, the association between different types of sports and lifespan is yet to be considered. Accordingly, we aimed to study this association in a large international cohort of former athletes using a robust linear regression model. We collected data on athletes from public sources, accumulating a total of 95,210 observations, 95.5% of which were accounted for by males. The dataset represented athletes born between 1862 and 2002 from 183 countries across 44 sports disciplines. We calculated the change in lifespan by measuring the difference in age between athletes and the corresponding reference populations, while accounting for variations caused by sex, year of death, and country. The results revealed that various sports impacted lifespan differently, with male athletes being more likely to experience benefits from sports than female athletes. Among male athletes, pole vaulting and gymnastics were linked to the highest extension in lifespan (8.4 years, 95% CI [6.8, 9.9] and 8.2 years, 95% CI [7.4, 9], respectively), while volleyball and sumo wrestling were the most negatively associated with lifespan (− 5.4 years, 95% CI [− 7, − 3.8]; − 9.8 years, 95% CI [− 11, − 8.6], respectively). The association between lifespan and popular team sports in males was positive for cricket, rowing, baseball, water polo, Australian rules, hurling, lacrosse, field hockey, minimal for rugby, canoeing and kayaking, basketball, gridiron football, and football (soccer), and negative for handball and volleyball. Racquet sports (i.e., tennis and badminton) exhibited a consistent and positive association in both male and female athletes, as shown by an extended lifespan of up to 5.7 years in males (95% CI [5, 6.5]) and 2.8 years in females (95% CI [1.8, 3.9]). Although lacking conclusive evidence, we theorize that the observed results may be attributed to the aerobic and anaerobic characteristics of each sport, with mixed sports yielding the maximum benefits for the lifespan. While results from female athletes should be cautiously interpreted, our study highlights the complex interplay between sports and lifespan and contributes to the growing body of knowledge on the multifaceted relationship between physical activity and human longevity.

**Graphical Abstract:**

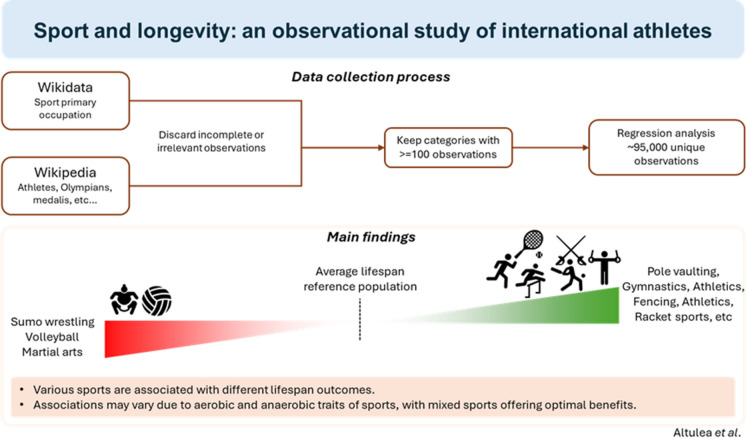

**Supplementary Information:**

The online version contains supplementary material available at 10.1007/s11357-024-01307-9.

## Introduction

Lifestyle plays a pivotal role in human longevity, and recent data suggest that modifiable factors, such as diet, psychosocial health, and physical activity, have a more profound impact on life expectancy than non-modifiable factors, such as genetics [1]. Regular physical activity has been proven to yield a range of physiological benefits that collectively contribute to improved overall health, including cardiorespiratory, skeletal muscle, and metabolic health [2]. Evidence from epidemiological research suggests that individuals performing various levels of physical activity show a significant reduction in the risk of developing age-associated diseases, including cardiometabolic diseases, certain types of cancer, osteoporosis, and depression [3], as well as a dose–response reduction in the risk of all-cause mortality [4], thus resulting in extended longevity.

Although numerous studies have demonstrated the benefits of physical activity on healthspan and lifespan [2], the association exhibits a non-linear pattern: while moderate activity is clearly advantageous compared to sedentary behavior, very vigorous exercise may have the opposite effects, leading to an increased occurrence of cardiovascular events (potentially in combination with undiagnosed cardiac abnormalities) and suboptimal survival rates in endurance-trained athletes [5–7]. Additionally, certain sports, such as boxing [8] and Gridiron football [9], have been associated with poor survival in males. A recent report based on the Finnish Twin Cohort found that leisure physical activity is associated with a lower risk of total mortality only in the short term compared to sedentary subjects, and it may only reflect a healthy phenotype rather than reducing mortality in the long term [10]. How long-term engagement in certain sports affects longevity remains to be fully explored, especially when considering the intensive training programs that athletes often adopt. Although it is clear that when elite athletes are analyzed together they have a lower mortality rate than the general population, the association between specific sports and longevity is much less studied [11]. Notably, some of the reports that do include sport-specific longevity numbers underscore the potential adverse impact of certain types of sports on lifespan [8, 9, 11]

To explore the association between sports and lifespan further, we included publicly available data from a cohort of international athletes. Based on previous literature, our working hypothesis was that participation in sports is associated with a certain degree of lifespan extension, with endurance-associated sports likely yielding greater extension than sports focusing on resistance training. Understanding this association is critical for comprehending the collective benefits of participating in sports on human health and longevity, independent of other factors that may be linked to longevity and may provide valuable information for sportspersons to make informed decisions about choosing activities that positively contribute to better health and longevity.

## Methods

### Data extraction and study population

**Fig. 1:**
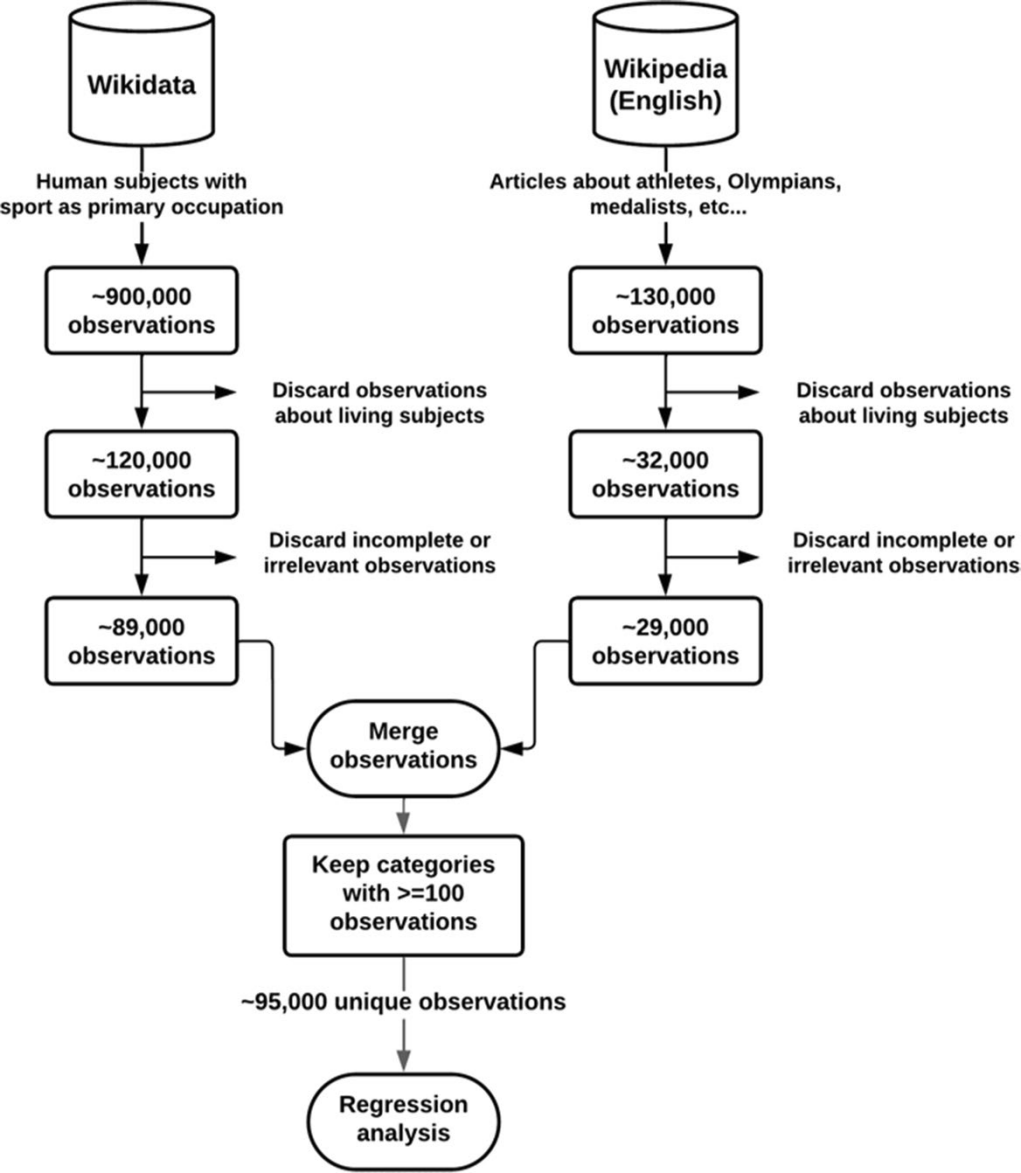
Flow chart depicting the data collection process

We collected data on former elite athletes from publicly available sources, primarily Wikidata and Wikipedia. We searched Wikidata for every data item that is an instance of “Human” and with a reported “Sport” attribute. Similarly, English Wikipedia was searched for articles containing the keywords athlete, Olympian, champion, or medalist. We filtered all observations based on the availability of demographic data, including year of birth, year of death, sex, country, and the primary sport practiced by the athlete. Certain observations were filtered out based on the reported doping use, Paralympics sports, or non-demanding sports (such as billiards and bowling). Observations were further filtered based on reported causes of death, excluding observations that cited accidental or unnatural deaths. Observations with missing values, from countries that were not found in the reference, or belonging to a sport category with less than 100 total observations per sex were excluded from the analysis (see Fig. 1 for an overview of the process, or Supplementary Methods for more details).

### Classification of sports into categories

We manually classified sports into distinct categories, with a few exceptions, where grouping was possible or needed to attain the given sample number per category. In such cases, two or more related sports were merged into a broader category representing the principles or skills of sports. We manually categorized the following: any variants of -athlon sports, such as decathlon and pentathlon, under “-athlon sports”; high jump, long jump, and other related jumping sports that are part of track and field, under “jumping sports”; judoka, taekwondo, judo, and other forms of martial arts, under “martial arts”; tennis and badminton under “racquet sports”; shooting and archery under “targeting sports”; all sports involving horse riding, under “equestrian sports”; ice hockey and bandy under “stick sports (ice)”; hurling, lacrosse, and field hockey under “Stick sports (field)”; and shot put and discus throw under “throwing sports.” Participants who took part in multiple track and field activities were categorized under the “mixed track and field” category. Note that the category “football” refers to soccer, while “Gridiron football” refers to North American football. (See the Supplementary Methods section for more details on the classification.)

### Life expectancy reference

We used the “life expectancy” data from the World Bank [12] as a reference to estimate the changes in the lifespan of athletes. The dataset contains life expectancy data for both males and females across 236 territories from 1960 through 2021. We used this reference as the basis for calculating the age delta for each observation in the study population. Age delta (age Δ) is our measure of lifespan extension and is defined as the numerical difference in years between the age of an athlete and the age of the corresponding general population of the same sex and country at a given year of death. A positive age Δ indicates a longer lifespan than that of the reference population. This metric allows us to acquire sex-, year of death-, and country-normalized estimates that reflect the change in the lifespan of athletes from the general population.

### Regression and statistical analyses

To address the outliers, influential observations, and the negative skewness that is usually associated with longevity data, we employed univariable robust regression using the ‘lmrob’ function from the robustbase R package [13], with the parameter “KS2014.” The model assessed the relationship between the change in lifespan (age Δ) and the type of sport. The dependent variable, age Δ, was regressed on the independent variable, type of sport, which was categorically coded. We implemented a zero-intercept model based on the assumption that in the absence of sports, the change in lifespan would be expected to be zero, indicating a zero change from the population’s life expectancies. We adopt this assumption because of the lack of a reference level for the independent variable. Since age Δ, the change in lifespan, was calculated based on estimates that account for differences in sex, year of death, and country, no adjustments for confounders were made. Owing to the underrepresentation of female athletes in the dataset, we chose to run the model separately for each sex. Regression results are presented as a forest plot (generated using the ‘Forestplot’ R package [14]), showing the regression coefficients and 95% confidence intervals. A test was considered statistically significant when the *p*-value was less than the chosen α level of 0.05. All analyses were performed using R version 4.2.3.

## Results

### Baseline characteristics of the study population

Table 1 presents the characteristics of the study population. The cohort comprised 95,210 athletes across 44 sport categories and 183 countries. Years of birth ranged between 1862 and 2002, whereas years of death ranged from 1960 to 2021 (the limits of the reference). Male athletes accounted for 95.5% of the total observations, with a median age increase of 3.1 years compared with the reference values, while female athletes accounted for 4.5% of the observations, with a median age decrease of − 0.7 years. Among the total observations, 63.4% originated from the nine countries listed in Table 1, indicating a reporting bias in the data source. Observations of male athletes belonged to 182 countries, while female athletes belonged to 101 countries. Data for female athletes were absent from Asia, Africa, Eastern and Central Europe, Middle East, and Central and South America (data not shown). Among the nine countries listed in Table 1, only Germany, Sweden, and the UK demonstrated a consistent median age increase between male and female athletes. Football accounted for the majority of observations, followed by baseball, gridiron football, cricket, and cycling.

**Table 1:** Characteristics of the study population across sex, sport, and country of origin. Data are presented as *n* (%) or median [interquartile range]

	Median age Δ (years)	
	Males	Females
Sex		
Males, *n* = 90,931 (95.5%)	3.10 [− 6.14, 10.40]	–
Females, *n* = 4279 (4.5%)	–	0.70 [− 9.70, 7.20]
Sport		
-athlon sports	5.90 [− 1.70, 12.90]	^‡^
Australian rules	4.40 [− 2.70, 10.29]	^‡^
Baseball	4.90 [− 4.00, 11.80]	0.80 [− 6.40, 6.70]
Basketball	2.70 [− 8.11, 10.00]	− 4.50 [− 18.20, 4.10]
Bobsledding/luging	0.50 [− 9.80, 8.20]	^‡^
Boxing	0.30 [− 9.38, 7.30]	^‡^
Canoeing/kayaking	2.70 [− 7.23, 9.54]	^‡^
Cricket	6.40 [− 1.80, 13.00]	− 3.40 [− 13.05, 4.55]
Curling	1.85 [− 8.88, 9.50]	^‡^
Cycling	3.33 [− 6.20, 10.30]	^‡^
Diving	6.29 [− 3.52, 11.90]	2.10 [− 7.60, 8.25]
Equestrian sports	5.09 [− 4.10, 12.50]	^‡^
Fencing	7.21 [− 1.10, 14.55]	1.60 [− 6.60, 8.70]
Football	1.50 [− 7.42, 8.40]	^‡^
Golf	4.48 [− 4.37, 12.80]	5.05 [− 3.65, 10.37]
Gridiron football	2.00 [− 7.91, 9.70]	^‡^
Gymnastics	8.80 [2.34, 14.40]	0.80 [− 9.80, 7.30]
Handball	− 2.10 [− 11.50, 6.90]	^‡^
Hurdling	6.02 [− 3.25, 13.19]	^‡^
Jumping sports	5.34 [− 3.33, 11.99]	1.30 [− 10.44, 6.95]
Martial arts	− 2.10 [− 14.15, 8.43]	^‡^
Mixed track and field	6.70 [− 2.35, 13.49]	0.00 [− 15.43, 7.12]
Mountaineering	− 1.59 [− 27.57, 10.15]	^‡^
Pole vaulting	9.00 [2.15, 14.73]	^‡^
Racewalking	5.55 [− 0.25, 12.00]	^‡^
Racquet sports	6.39 [− 2.87, 14.10]	4.09 [− 5.52, 10.80]
Rowing	5.74 [− 2.90, 11.91]	^‡^
Rugby	2.64 [− 6.12, 9.70]	^‡^
Running	5.20 [− 2.96, 11.96]	^‡^
Sailing	5.22 [− 2.95, 12.60]	^‡^
Skating	4.26 [− 5.65, 12.57]	1.40 [− 6.73, 7.73]
Skiing	5.30 [− 3.60, 12.00]	− 1.15 [− 10.25, 5.90]
Sprinting	5.80 [− 1.49, 13.18]	− 0.60 [− 10.63, 6.60]
Stick sports (field)	4.16 [− 4.97, 12.00]	^‡^
Stick sports (ice)	0.51 [− 10.20, 8.15]	^‡^
Sumo	− 9.20 [− 20.09, − 0.32]	^‡^
Swimming	2.70 [− 7.51, 11.20]	− 0.21 [− 8.60, 6.50]
Table tennis	0.20 [− 7.70, 9.22]	− 4.20 [− 14.15, 5.62]
Targeting sports	7.20 [− 2.17, 14.41]	^‡^
Throwing sports	2.93 [− 6.17, 9.93]	0.70 [− 8.00, 5.90]
Volleyball	− 5.30 [− 17.06, 5.20]	^‡^
Water polo	4.10 [− 3.81, 11.40]	^‡^
Weightlifting	2.06 [− 8.68, 10.84]	^‡^
Wrestling	1.90 [− 10.40, 9.70]	^‡^
Top countries by observations		
USA	4.40 [− 5.10, 11.60]	1.35 [− 8.10, 7.90]
UK	3.30 [− 4.52, 9.60]	3.10 [− 6.10, 8.55]
Italy	1.46 [− 6.88, 8.16]	− 1.20 [− 10.20, 4.30]
France	4.10 [− 4.65, 10.50]	1.95 [− 10.62, 6.62]
Australia	3.30 [− 4.34, 9.60]	− 1.76 [− 13.10, 6.38]
Germany	0.94 [− 8.70, 7.95]	1.10 [− 9.30, 6.60]
Canada	1.30 [− 8.49, 8.24]	− 3.10 [− 12.37, 6.16]
Sweden	3.80 [− 3.71, 9.60]	3.30 [− 6.06, 8.58]
Japan	− 4.56 [− 15.09, 3.53]	− 6.33 [− 21.26, 4.41]
Other	3.40 [− 6.81, 11.20]	− 0.06 [− 10.50, 6.92]

^‡^*n* < 100; excluded from the analysis

### Results of the regression analysis

**Fig. 2:**
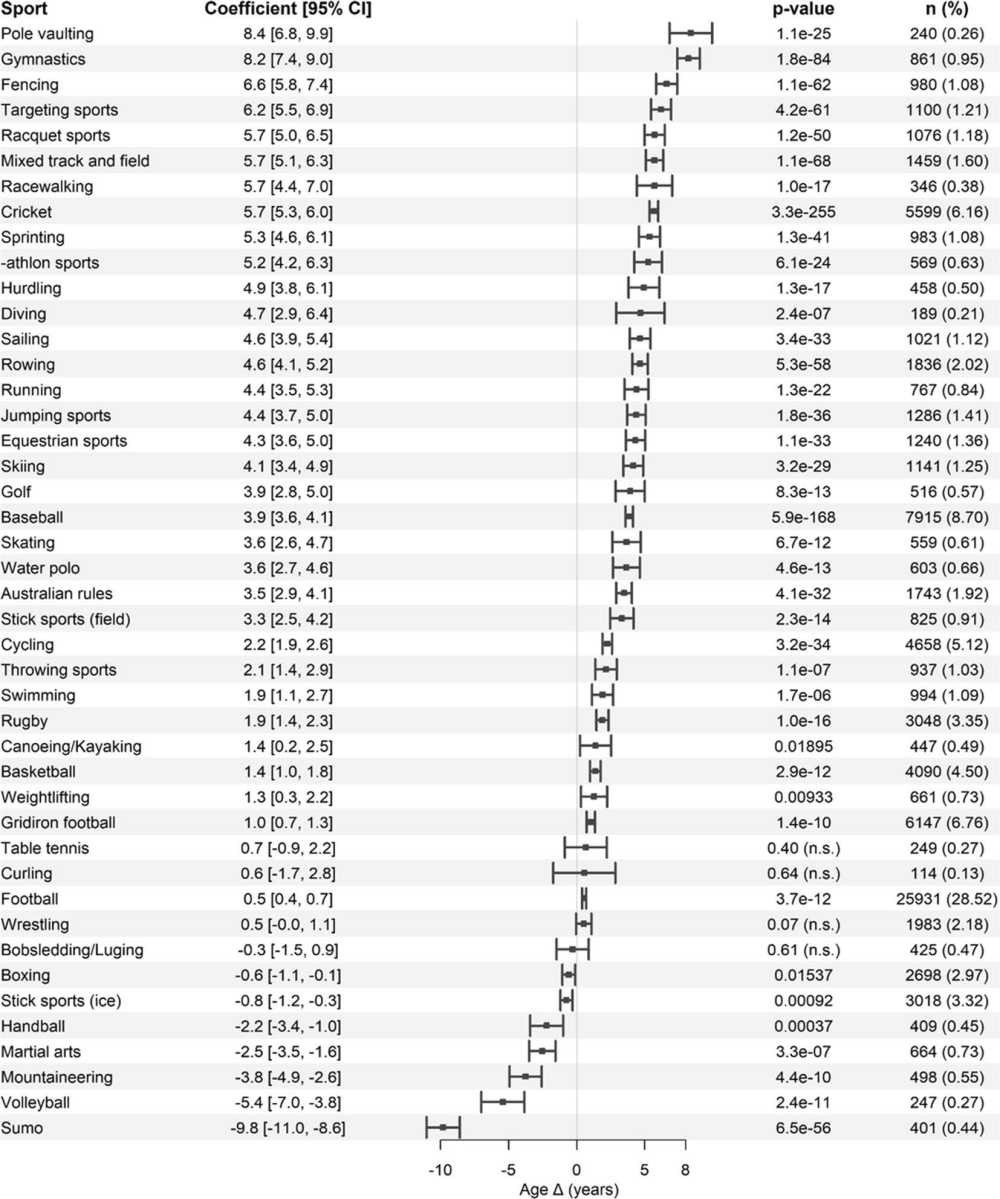
Association between various sports and changes in lifespan (age Δ) in male athletes. Coefficient values represent the average change in lifespan in years compared with the reference male populations. Percentage was calculated based on the total number of male athletes. Adjusted R-squared = 0.06216. n.s. indicates statistically non-significant results

The robust linear regression model revealed an overall positive association between various sports and Δ age in male athletes (Fig. 2). The adjusted *R*-squared value of 0.06216 suggests that approximately 6.2% of the variance in age Δ is accounted for by sport type. In terms of mean change in age in male athletes, pole vaulting showed the highest increase at 8.4 years, followed by gymnastics at 8.2 years, and sports associated with affluence, including fencing and targeting sports (e.g., archery), at 6 years. Several track and field sports, including racewalking, running, sprinting, hurdling, -athlon sports, and jumping sports, have been associated with an increase in lifespan, ranging from 4 to 5 years. Throwing sports of track and field were associated with a modest 2-year increase in age, suggesting an inherent characteristic within this category that yields a relatively lower age increase compared with the other track and field disciplines. No strong associations with lifespan were observed for table tennis, curling, football, wrestling, bobsledding/luging, boxing, or ice stick sports (e.g., ice hockey). Conversely, handball, martial arts, mountaineering, volleyball, and sumo wrestling are associated with a reduced lifespan, leading to a reduction of 2–9 years. All reported changes in age were relative to the reference male populations.

**Fig. 3:**
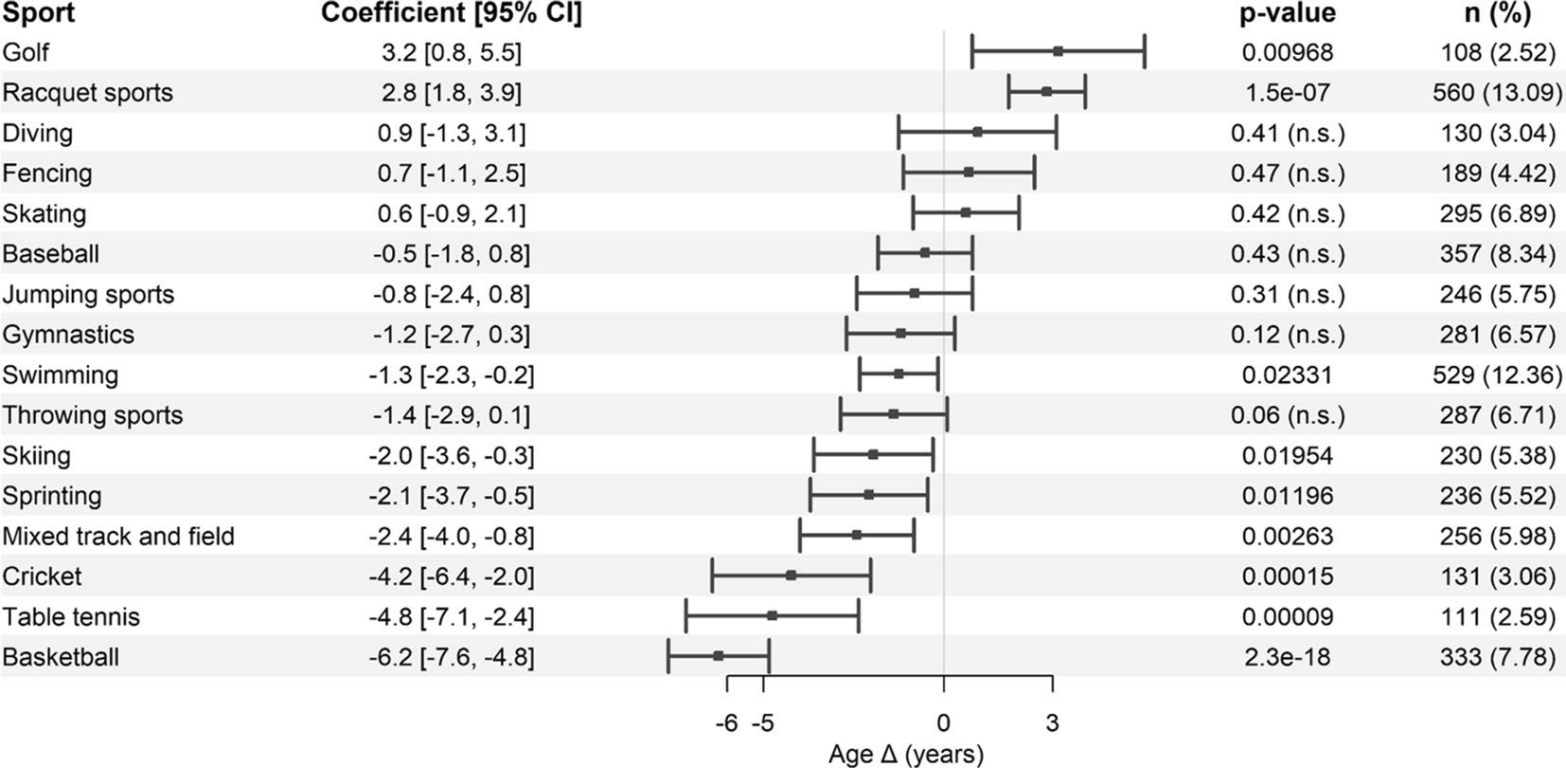
Association between various sports and changes in lifespan (age Δ) in female athletes. Coefficient values represent the average change in lifespan in years compared to the reference female populations. Compared with the male athletes, several categories were omitted because of the low number of observations. Percentage was calculated based on the total number of female athletes. Adjusted R-squared = 0.03529. n.s. indicates statistically non-significant results

The analysis revealed a few positive associations between sports and lifespan in female athletes, with most sports yielding negative changes ranging from − 1 to − 6 years (Fig. 3). Approximately 3.5% of the variance in age Δ in females is influenced by the type of sport, as indicated by the R-squared value of 0.03529. Among the categories examined, only golf and racquet sports exhibited a significant positive correlation with age, resulting in increments ranging from 2 to 3 years. Due to the limited sample size, the associations observed were weak and should be cautiously interpreted**.**

## Discussion

While the general positive impact of physical activity on longevity has been acknowledged in the literature [15–21], the association between various types of sports and lifespan has been minimally addressed. In this study, we present regression analyses of this association, uncovering significant changes in the lifespan of former elite athletes compared to reference populations across different types of sports. The analysis also revealed a discrepancy between male and female athletes, with males experiencing greater benefits from professional sports than females. Understanding the effects of participation in professional sports on lifespan provides valuable insights into the complex interplay between physical activity and human longevity.

Our results suggest that participation in sports has a mostly favorable impact on the lifespan of male athletes, with some exceptions. This is consistent with the findings from the narrative review by Teramoto et al. [11] who pooled studies that examined longevity in elite athletes and assessed whether specific types of sports are associated with greater impact on lifespan and survival rates. In the review, the authors found that, compared with the general population, endurance and mixed sports showed increased survival rates, whereas anaerobic sports showed inconsistent results. While most of the pooled studies dealt with males, a few studies have highlighted the benefits of such sports in female athletes. From our analysis, as shown in Fig. 2, the sports exhibiting the highest increase in lifespan in males are pole vaulting and gymnastics, which are predominantly mixed sports that incorporate anaerobic and aerobic components. Conversely, Sarna et al. [22] examined the impact of sports on lifespan in highly trained athletes and found that aerobic sports, such as running and skiing, were associated with greater increases in lifespan than mixed and anaerobic sports. The discrepancy between our results and their results is likely due to the limited number of sports they tested as well as the different categorization criteria. Nevertheless, we observed a weak negative association between lifespan and boxing, modest associations with football and basketball, and a strong association with running. We propose that mixed sports yield a greater extension in lifespan than predominantly aerobic or anaerobic sports because engaging in such sports will result in (1) improved cardiorespiratory health, reduced blood pressure, body weight, percent body fat, and improved insulin sensitivity, which are common benefits associated with endurance training [20, 23], and (2) improved muscle mass [24, 25], slower decline in aerobic capacity [26], improved neuromuscular function [27], and higher bone mineral density than endurance athletes [26, 28] as well as protection against sarcopenia and falls [29], which are some of the phenotypes associated with resistance training.

Unsurprisingly, combat sports, such as martial arts and sumo wrestling, exhibited a negative association with the lifespan of males (Fig. 2). This could be partially attributed to the inherent risk of traumatic injuries that these sports subject their practitioners to [30]. Moreover, the extreme diet imposed by sumo sports may further exacerbate its negative impact on the lifespan of sumo wrestlers. Neither wrestling nor boxing showed a strong association with lifespan, which is consistent with the results of Sarna et al. [22]. The differences between martial arts and wrestling underline the presence of alternative factors that may influence the negative association between combat sports and lifespan beyond traumatic injuries.

Strikingly, volleyball appeared to be negatively associated with lifespan in both male and female athletes (Fig. 2 and Fig. 3). However, the cause of this association remains unclear. We hypothesize that the physical trauma that volleyball players are exposed to [31] may instigate severe skeletomuscular stress, which impacts healthspan, and, in the long term, lifespan. A comparable rationale can be applied to handball, which also showed a negative association and may involve similar sporting characteristics to volleyball. Nevertheless, establishing a causal relationship in light of these observations poses a challenge due to the complexity of sports and the nature of this study.

The observed results from our analyses can likely be attributed to various molecular changes, consistent with the existing literature that highlights the beneficial effects of physical training on epigenetic age markers, circulating complements, cytokines, telomere length, and DNA damage [32–34]. For instance, Al-Muraikhy et al. [32] suggested that high-intensity endurance athletes have lower levels of specific complements associated with inflammation, oxidative stress, and cellular aging compared to low- and moderate-intensity endurance athletes. Additionally, Sellami et al. [33] showed that high-intensity endurance athletes present longer telomeres and higher levels of both pro- and anti-inflammatory cytokines than athletes performing low- and moderate-intensity endurance sports. Alternatively, in another review, Sellami et al. [35] reported that, while aerobic and/or endurance training appears most promising for conserving telomere length, low-volume resistance training with moderate intensity also proves beneficial for the maintenance of telomere length in older adults. Moreover, they argued that extremely long-duration endurance training, such as ultra-distance running, when performed for long periods, may shorten telomeres due to accelerated oxygen radical generation and oxidative stress. Borghini et al. [36] similarly found that acute exposure to an ultra-distance endurance trail race results in telomere shortening. Conversely, the reports addressing athletes’ lifespan and telomere length are inconsistent. Experimental evidence suggests that endurance athletes have longer telomeres than non-athletes [37–40]. Meta-analyses also found that master athletes, primarily endurance athletes, exhibit longer telomeres compared to age-matched non-athletes [41, 42]. However, a study demonstrated that endurance and interval training increase telomerase activity and telomere length, while resistance training does not [43]. Furthermore, a meta-analysis revealed that only high-intensity interval training significantly enhances telomere length compared to resistance or aerobic training [44]. This inconsistency might result from the original studies either not comparing various training types, ignoring the effect of sex, neglecting to consider the duration of exercise, or reporting telomere length from different sources, e.g., leucocytes or muscles.

Another rationale for extending the lifespan of athletes is hypoxia. This can occur as a result of exercise-induced hypoxemia, altitude training, or intermittent hypoxia training approaches. While the understanding of exercise-induced hypoxemia is an evolving area of research [45], both chronic and intermittent hypoxia have been shown to extend the lifespan of model organisms [46–49]. The beneficial effects of hypoxia are likely explained by its hormetic nature (i.e., the ability to induce mild stresses that allow an organism to increase its resistance to more extreme stresses later in life) and its influence on metabolic and mitochondrial reprogramming, antioxidant signaling, and inflammation [50–52]. Therefore, the impact of hypoxia should be considered, as it may partially explain the observed lifespan extension without directly reflecting the underlying impact of the sport itself.

The current analysis revealed a mostly negative association between sports and lifespan among female athletes, with only a few sports showing a positive association. This suggests a relatively diminished overall benefit of professional sports for females compared with males. These results contradict the findings of a few studies reviewed in [11], which showed a reduced mortality ratio in female athletes compared to that in females from the general population. Conversely, Armstrong et al. [53] observed that women who engage in vigorous physical activity every day are at a higher risk of developing cardiovascular events, including coronary heart disease, cerebrovascular disease, and venous thromboembolism, than women who engage in the same activity two to three times a week. Although the exact cause of the lack of advantages of professional sports on lifespan in female athletes remains uncertain, we propose a few non-mutually exclusive hypotheses. First, the general female population exhibits a consistently longer life expectancy than males [12], suggesting the possibility that they may have already reached the maximum attainable lifespan extension at a given time point, and engaging in prolonged physical activity may subject them to stresses that elevate their risk for developing adverse outcomes. Second, it is conceivable that females may derive benefits from moderate physical activity, as opposed to the strenuous activities associated with professional athletic lifestyles [53]. Third, the dietary habits associated with certain sports might predispose female athletes to compromised metabolic health compared with the diets adhered by the general female population. Fourth, the induction of anabolic hormones in response to various types of physical activities in women [54] raises the possibility that chronic exposure to such hormones may adversely affect lifespan in the long term. Fifth, the disparity in behavioral patterns between the sexes could partially influence the observed results. Males from the general population tend to engage in morbid behaviors, such as smoking and alcohol consumption, which severely impact their life expectancy compared with females who are, on average, less likely to engage in such behaviors [55–57]. We propose that participation in sports may prompt males to minimize such habits, leading to a more profound impact of sports on their lifespan. For instance, Sarna et al. [22] reported that between 11 and 17% of male athletes they studied were actively smoking, whereas in the reference population, this was 26%. On the other hand, females are less likely to adopt morbid behaviors; thus, engaging in professional sports might not confer substantial benefits to their lifespan. Nevertheless, the notably small sample size of female athletes limits our ability to draw definitive conclusions about the association between sports and lifespan. This highlights the historical underrepresentation of females in professional sports throughout the study period.

A cohort of international athletes represented a unique study population for our investigation because of their distinctive lifestyles, which may include extensive training regimens and specialized dietary demands. Using this population enables us to incorporate various factors which are typically perceived to impact lifespan [58, 59], such as diet, and physical activity (including the impact of endurance and resistance training), as well as the social environment that accompanies team sports. For instance, certain sports, such as long-distance cycling, require high-calorie diets [60], which are conventionally associated with a negative impact on lifespan in model organisms [61]. However, our analysis revealed that cycling was associated with a positive change in the lifespan of male athletes. Moreover, it is possible that the competitive careers of athletes, though limited by retirement, provide a unique perspective into the long-term consequences of engaging in an athletic lifestyle during a specific period of one’s life, thus highlighting the effects of the post-retirement transition of athletes on lifespan.

This study has several limitations that should be acknowledged. Many of the collected observations were from athletes from Europe and North America (see Table 1, “Top countries by observations”), indicating a reporting bias in the source of data. The overrepresentation of these regions may limit the generalizability of our findings to a more global context. Moreover, we did not specifically screen “elite” athletes using specific cutoff values. However, all sportspersons competing at the national or international level, Olympic or otherwise, were included in the study, which we believe represents a highly trained athlete population. Notably, only athletes who attained sufficient popularity to be documented in the public domain were included in this study. Furthermore, the small sample size of female athletes significantly hinders our ability to reach conclusive findings regarding the association between sports and lifespan. Finally, even though the primary measure of the study, age Δ, mitigates geographical variations by normalizing the age of athletes to the corresponding general population, it does not consider the variation in life expectancy caused by ethnicity or socioeconomic status. Ethnicity, education, and socioeconomic status not only contribute to distinct patterns of aging in the population [62–64], but also limit the access of certain individuals to certain sports, often making the latter exclusive to those with higher socioeconomic levels. Failure to incorporate these factors into our model may have led to an incomplete understanding of the relationship between lifespan and sports for athletes across diverse ethnic backgrounds and socioeconomic statuses. Regrettably, the data sources lacked information on these factors, precluding our ability to address this issue.

In summary, this study established an association between various types of sports and the lifespan of a diverse cohort of international athletes. The impact on lifespan varies across sports, with notable differences between male and female athletes. While male athletes experienced a mostly favorable lifespan extension, with an increment of up to 8 years across different sports, female athletes had limited and scarce data, thus limiting our ability to draw definitive conclusions regarding this association.

## Supplementary Information

Below is the link to the electronic supplementary material.

Supplementary file1 (DOCX 22 KB)

## Data Availability

The dataset and Python scripts used in this study are available from the corresponding author upon request.
