# Supplementary material for: Sport and longevity: an observational study of international athletes
Source: GeroScience. 2024 Aug 12;47(2):1397–409. doi: 10.1007/s11357-024-01307-9 (PMC11979035; doi:10.1007/s11357-024-01307-9)
Supplement: Supplementary file 1 — Supplementary file1 (DOCX 22 KB) [file 11357_2024_1307_MOESM1_ESM.docx]

**Supplementary Methods**

We chose to collect data from two sources to check the consistency of the collected data.

*Data collection from Wikipedia*

1. Obtain an offline copy of English Wikipedia
2. Iterate over every article and check for the following keywords (case-insensitive):
   Athlete, Sprinter, Athletics, Athletic, Bodybuilder, Wrestler, Swimmer, Champion, Athletics, Medalist, Olympian, Olympic, Olympics, Cyclist, Tennis, Soccer, Basketball, Volleyball, Wrestler, Boxer, Gymnast, Diver, Skier, Snowboarder, Surfer, Skateboarder, Archer, Fencer, Martial artist, Triathlete, Rower, Weightlifter, Powerlifter, Javelin thrower, Shot putter, Pole vaulter, Discus thrower, High jumper, Long jumper, Racewalker, Taekwondo
3. Only keep articles about dead individuals (RegEx: \\bwas a\\b|\\bwas an\\b).
4. Extract the type of sport that the athlete engaged in, as well as the country of origin, from the first sentence in the article. (E.g., *Muhammad Ali was an American professional boxer and activist.*)
5. Determine the sex of an athlete using RegEx from the first sentence in the article.
6. Extract year of birth and year of death using RegEx (r"\([^\)]*?(\d{4})[^\d{4}\)]*?[-—−–].*?(19\d{2}|20\d{2})[^\d{4}\)]*?\)")
7. Determine the cause of death if mentioned, from checking all available versions of the articles. Some causes of death are only mentioned in non-English Wikipedia. We used an offline translation tool to translate sentences to english.

*Data collection for Wikidata*

1. Obtain an offline copy of Wikidata
2. Iterate over all items that are instances of Human (P31 == Q5) and have a reported sport (P641 != NULL)

*For all observations*

1. Exclude cases that mention doping, substance use, anabolic steroids.
2. Exclude incomplete cases based on: sex, year of birth, year of death, nationality, and sport practiced. Athletes with known multiple sports were excluded from wikipedia.
3. Exclude causes of death that reflect unnatural death. The following keywords were used for the exclusion:

crash, crashing, accident, incident, car, plane, airplane, traffic, collision, train, aircraft, motorcycle, accidentally, on the battlefield, communist, socialists, chain broke, suffocation during a severe epileptic seizure, swallowing chloral hydrate, construction site, house fire, foreset fire, large fire, injuries she sustained in a fire, a fire broke out, bullet, gunshot, shot, rifle, gunfire, gun, revolver, killed, executed, extermination, exterminated, execution, massacres, massacre, assassinated, assassination, stabbed, stabbing, murdered, murder, slaughtered, crushed, protests, shootout, choking, wounded, bombing raid, terrorists, bomb, stab wounds, fighting, theft, mob, childbirth, pregnancy complications, Holocaust, imprison, poisoned, poisoning, malnutrition, starvation, dehydration, sentenced to death, overdose, overdosing, driving drunk, drug, drugs, intoxication, toxicity, doping, anabolic steroids, fell from, falling on, fall, fell, falling into, falling down, struck, blow, injuries due to a fall, avalanche, suicide, jumping from, self-inflicted, hanging, shooting himself, shooting herself, electrocuted, electrocution, high power line, cable, exploded, explosion, grenade, drowned, drowning, broken neck, anorexia, sexual reassignment, intersex, surgery of reassignment of sex, poverty, alcoholism, hypothermia, syndrome, smoke, smoking, smoker, anaphylaxis, allergic, allergy, meningitis, polio, tetanus, typhus, tuberculosis, spanish flu, h1n1, asthma, pneumonia, gangrene, Covid, SARS-CoV, pandemic, seizure.

1. Filtered out paralympians
2. Manually correct some incorrect age information for observations with negative age or age that is below 18 years.
3. Calculate age delta based on reference. Athletes who died before 1960 or after 2021 were excluded.

*Regression analysis*

The following robust regression model was run for each sex separately.

(age delta ~ 0 + type of sport)

Where **age delta** is a continuous variable representing how many years a person lived more or less compared with a reference population; **type of sport**: a categorical variable with several levels representing the manually classified sports (check Table 1 from the main article to see the levels). No reference level was set for this variable, and a forced zero intercept was used to account for the lack of a reference level. We adopted a zero intercept assumption based on the expectation that in the absence of all sports, the change in lifespan (age delta) would be equal to zero. Specifically, when an individual refrains from participating in any form of elite sporting program, their age would then be presumed to align with the population average, resulting in a zero age delta. We ran the model for each sex due to the underrepresentation of females. Additionally, since the age delta accounts for sex, country, and year of death, no variables were adjusted. For each category, a minimum of 100 observations were needed to be eligible for analysis.

Sport classification

| **Label** | **RegEx pattern** | **Segments** |
| --- | --- | --- |
| Sprinting | ^100 and 200 metres$ | 100 and 200 metres |
| Sprinting | track and field sprinter$ | track and field sprinter |
| Hurdling | track and field hurdler$ | track and field hurdler |
| Jumping sports | track and field high jumper$ | track and field high jumper |
| athletics | athletics$ | athletics |
| Mixed track and field | ^track and field$\|^track-and-field$ | track and field, track-and-field |
| Canoeing/Kayaking | ^canoeing and kayaking$ | canoeing and kayaking |
| (keywords containing 'and') | \\band\\b\|\\bund\\b | and, und |
| Rugby | \\brugby | rugby |
| Gridiron football | american football\|canadian football\|defensive lineman\|halfback\|defensive end\|defensive tackle\|gridiron football\|offensive lineman\|offensive tackle\|quarterback\|Football League running back | american football, canadian football, defensive lineman, halfback, defensive end, defensive tackle, gridiron football, offensive lineman, offensive tackle, quarterback, Football League running back |
| Australian rules | \\brules | rules |
| Sumo | \\bsumo | sumo |
| Skiing | \\bskier\|\\bskiing\|alpinist\|nordic combined | skier, skiing, alpinist, nordic combined |
| Equestrian sports | \\bequestr\|\\bhorse\|^polo$\|jockey | equestr, horse, polo, jockey |
| Jumping sports | \\bjump\|\\bjumper\|\\bjumping | jump, jumper, jumping |
| Throwing sports | \\bdiscus\|\\bshot put\|shot putter$\|shot put$\|\\bthrow\|diskos\|javelin\|hammer\|\\bdisk\\b | discus, shot put, shot putter, shot put, throw, diskos, javelin, hammer, disk |
| Hurdling | \\bhurdl | hurdl |
| Pole vaulting | \\bpole vault | pole vault |
| Racewalking | \\bracewalker\|\\bracewalking\|\\brace walker\|\\bkilometre walk\|20 kilometer walk$\|^walk$ | racewalker, racewalking, race walker, kilometre walk, 20 kilometer walk, walk |
| Running | \\brunner\|\\bmarathon\|distance$\|distance run\|running$\|maraton | runner, marathon, distance, distance run, running, maraton |
| Sprinting | \\bsprinter\|\\bmeters\|\\d\\d\\d m\|\\d\\d\\dm\|\\bmeter\|m.tres\|\\byard\\w?\\b\|\\bmeters\|\\bmetre\|\\bmetres\|100 m sprint\|^1500 m\|^200m$\|relay$\|sprint$\|sprinting$\|$sprints$\|^sprint | sprinter, meters, meter, m.tres, yard, meters, metre, metres, 100 m sprint, 1500 m, 200m, relay, sprint, sprinting, sprints, sprint |
| Softball | \\bsoftball | softball |
| Swimming | \\bswimming$\|\\bswimmer and olympic\|^swimmer\|breaststroke\|backstroke\|butterfly\|swimmer | swimming, swimmer and olympic, swimmer, breaststroke, backstroke, butterfly, swimmer |
| Sailing | sailor$\|sailing$\|\\bsailor and olympic medalist\|yachtsman | sailor, sailing, sailor and olympic medalist, yachtsman |
| Gymnastics | \\bgymnastics\|\\bgymnast\|acrobatics | gymnastics, gymnast, acrobatics |
| -athlon sports | \\bpentathlete\|\\bdecathlon\|\\bdecathlete\|\\bpentathlon\|\\bbiathlon | pentathlete, decathlon, decathlete, pentathlon, biathlon |
| Basketball | \\bbasketball | basketball |
| Volleyball | \\bvolleyball | volleyball |
| Skating | \\bskater\|\\bskating | skater, skating |
| Baseball | \\bbaseball\|\\boutfielder\|pitcher$\|catcher$\|infielder$ | baseball, outfielder, pitcher, catcher, infielder |
| Football | \\bsoccer\|\\bgoalkeeper\|\\bmidfielder\|\\bplayed as a forward\|\\bstriker\|\\bdefender\|association football | soccer, goalkeeper, midfielder, played as a forward, striker, defender, association football |
| Boxing | \\bboxer\|\\bboxing\|kickbox | boxer, boxing, kickbox |
| Rowing | \\brower\|\\browing\|coxswain | rower, rowing, coxswain |
| Cycling | \\bcyclist\|\\bbicycle\|\\bcycling\|\\broad and track cyclist\|^racing$\|\\bcycle | cyclist, bicycle, cycling, road and track cyclist, racing, cycle |
| Table tennis | \\btable tennis | table tennis |
| Racquet sports | \\btennis\|\\bbadminton | tennis, badminton |
| Stick sports (ice) | \\bice hockey\|^bandy$ | ice hockey, bandy |
| Stick sports (field) | hurling$\|lacrosse\|field hockey | hurling, lacrosse, field hockey |
| Cricket | \\bcricketer\|^cricket$ | cricketer, cricket |
| Targeting sports | \\bshooter\|\\barcher\|\\bshooting\\b | shooter, archer, shooting |
| Fencing | \\bfencer\|\\bfencing | fencer, fencing |
| Handball | \\bhandball | handball |
| Martial arts | judoka\|martial\|taekwondo\|judo\|sambo\|Muay Thai\|karate\|aikido | judoka, martial, taekwondo, judo, sambo, Muay Thai, karate, aikido |
| Weightlifting | \\bpowerlift\|\\bweightlift | powerlift, weightlift |
| Wrestling | \\bwrestler\|\\bwrestling\|\\bweight\\b\|men's freestyle\|^freestyle\|^featherweight freestyle$\|^flyweight\|heavyweight\|^middleweight$\|^lightweight$\|welterweight | wrestler, wrestling, weight, men's freestyle, freestyle, featherweight freestyle, flyweight, heavyweight, middleweight, lightweight, welterweight |
| Bodybuilding | \\bbodybuild | bodybuild |
| Curling | \\bcurler\|\\bcurling | curler, curling |
| Golf | \\bgolf | golf |
| Mountaineering | mountaineering\|rock climbing | mountaineering, rock climbing |
| Water polo | \\bwater polo | water polo |
| Diving | \\bdiver\|\\bdiving | diver, diving |
| Bobsledding/Luging | \\bbobsledder\|\\bbobsledding\|\\bluge\|bobsleigh | bobsledder, bobsledding, luge, bobsleigh |
| Canoeing/Kayaking | \\bcanoer\|\\bcanoeist\|\\bcanoeing\|canoeing and kayaking\|kayaking | canoer, canoeist, canoeing, canoeing and kayaking, kayaking |
| Mixed track and field | \\athlete | athlete |
